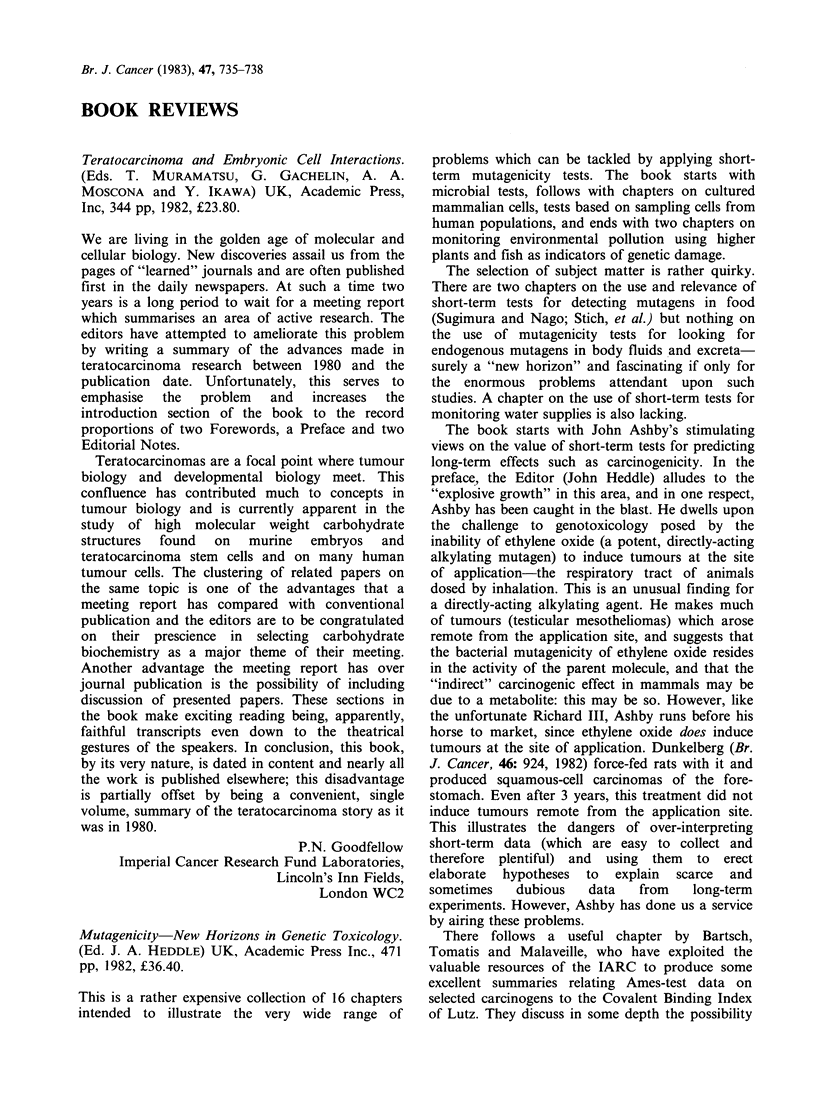# Teratocarcinoma and Embryonic Cell Interactions

**Published:** 1983-05

**Authors:** P.N. Goodfellow


*Br. J. Cancer* (1983), **47**, 735–738

### BOOK REVIEWS

*Teratocarcinoma and Embryonic Cell Interactions.* (Eds. T. MURAMATSU, G. GACHELIN, A. A. MOSCONA and Y. IKAWA) UK, Academic Press, Inc, 344 pp, 1982, £23.80.

We are living in the golden age of molecular and cellular biology. New discoveries assail us from the pages of "learned" journals and are often published first in the daily newspapers. At such a time two years is a long period to wait for a meeting report which summarises an area of active research. The editors have attempted to ameliorate this problem by writing a summary of the advances made in teratocarcinoma research between 1980 and the publication date. Unfortunately, this serves to emphasise the problem and increases the introduction section of the book to the record proportions of two Forewords, a Preface and two Editorial Notes.

Teratocarcinomas are a focal point where tumour biology and developmental biology meet. This confluence has contributed much to concepts in tumour biology and is currently apparent in the study of high molecular weight carbohydrate structures found on murine embryos and teratocarcinoma stem cells and on many human tumour cells. The clustering of related papers on the same topic is one of the advantages that a meeting report has compared with conventional publication and the editors are to be congratulated on their prescience in selecting carbohydrate biochemistry as a major theme of their meeting. Another advantage the meeting report has over journal publication is the possibility of including discussion of presented papers. These sections in the book make exciting reading being, apparently, faithful transcripts even down to the theatrical gestures of the speakers. In conclusion, this book, by its very nature, is dated in content and nearly all the work is published elsewhere; this disadvantage is partially offset by being a convenient, single volume, summary of the teratocarcinoma story as it was in 1980.

P.N. Goodfellow
Imperial Cancer Research Fund Laboratories,
Lincoln's Inn Fields,
London WC2